# Clinical features, anger management and anxiety: a possible correlation in migraine children

**DOI:** 10.1186/1129-2377-14-39

**Published:** 2013-05-07

**Authors:** Samuela Tarantino, Cristiana De Ranieri, Cecilia Dionisi, Monica Citti, Alessandro Capuano, Federica Galli, Vincenzo Guidetti, Federico Vigevano, Simonetta Gentile, Fabio Presaghi, Massimiliano Valeriani

**Affiliations:** 1 Division of Neurology, Headache Centre, Ospedale Pediatrico Bambino Gesú, IRCCS, Rome, Italy; 2 Unit of Clinical Psychology Ospedale Pediatrico Bambino Gesù, IRCCS, Rome, Italy; 3 Faculty of Medicine and Psychology, “La Sapienza” University of Rome, Rome, Italy; 4 Headache Science Centre of the IRCCS “National Institute of Neurology C. Mondino” Foundation, Pavia, Italy; 5 Center for Sensory-Motor Interaction, Aalborg University, Aalborg, Denmark

**Keywords:** Migraine, Anger, Anxiety, Children

## Abstract

**Background:**

Psychological factors can increase severity and intensity of headaches. While great attention has been placed on the presence of anxiety and/or depression as a correlate to a high frequency of migraine attacks, very few studies have analyzed the management of frustration in children with headache. Aim of this study was to analyze the possible correlation between pediatric migraine severity (frequency and intensity of attacks) and the psychological profile, with particular attention to the anger management style.

**Methods:**

We studied 62 migraineurs (mean age 11.2 ± 2.1 years; 29 M and 33 F). Patients were divided into four groups according to the attack frequency (low, intermediate, high frequency, and chronic migraine). Pain intensity was rated on a 3-levels graduate scale (mild, moderate and severe pain). Psychological profile was assessed by Picture Frustration Study test for anger management and SAFA-A scale for anxiety.

**Results:**

We found a relationship between IA/OD index (tendency to inhibit anger expression) and both attack frequency (r = 0.328, p = 0.041) and intensity (r = 0.413, p = 0.010). When we analyzed the relationship between anxiety and the headache features, a negative and significant correlation emerged between separation anxiety (SAFA-A Se) and the frequency of attacks (r = −0.409, p = 0.006). In our patients, the tendency to express and emphasize the presence of the frustrating obstacle (EA/OD index) showed a positive correlation with anxiety level (“Total anxiety” scale: r = 0.345; p = 0.033).

**Conclusions:**

Our results suggest that children suffering from severe migraine tend to inhibit their angry feelings. On the contrary, children with low migraine attack frequency express their anger and suffer from separation anxiety.

## Background

The role of affective factors in the experience of pain has received a great deal of attention over the past few decades. While several studies investigated the relationship between depression, anxiety and headache in both adult [1-3] and paediatric age [3,4], only a few reports dealt with anger.

Studies on chronic pain syndromes suggested that the way of expression/management of anger has an important effect on disease course and impact [5-7]. In particular, failure to express one’s own anger is related to more intense and frequent pain [5,6,8]. On the other hand, patients with chronic pain conditions show a significantly greater tendency, as compared to healthy subjects, to inhibit anger [5,9,10]. It is possible that the experience of long-lasting persistent pain leads to anger inhibition. Thus, a vicious circle can be established in which anger is inhibited by chronic pain and retained anger can increase affective component of pain.

That the relationship between anger and headache has been only rarely explored could be due to the complexity of defining anger and related constructs. Level of emotional intensity and physiological arousal related to anger show a large inter- and intra-individual variability [11]. Not only the frequency of anger expression, but also the way one’s own anger appears can be extremely variable. Anger expression refers to the usual models of expressing anger feeling. Three different components may be considered: 1) internalised anger, 2) externalised anger, and 3) anger control. Internalised anger (anger-in) reflects the tendency to suppress angry feeling. It occurs when an individual does not express his own anger outwardly, but inwardly, thus feeling considerable internal stress. In contrast, externalised anger (anger-out) leads to aggressive behaviour, such as physical or verbal acts against objects or persons in the environment. Lastly, anger-control refers to the ability to monitor and prevent the experience of anger [11].

Adults suffering from headache typically have a significant impartment in their ability to control and express angry feelings, as compared to healthy subjects [12-16]. Possible relationship between anger expression and severity of headache (frequency and intensity of the attacks) are scarce [13,15-17].

In children, data investigating a possible link between anger and headache are even fewer. To the best of our knowledge, only three studies described the role of anger in children suffering from headache and they did not use specific or standardized tools to assess the anger management style [18-20]. Moreover, in only one study [19] the ICHD-II diagnostic criteria [21] were considered.

It has been reported that migraine patients experience anxiety and/or guilt after expressing anger feeling [22]. However, the relationship between anxiety and anger has rarely been investigated in headache patients and no study was conducted in a pediatric population [12,16].

In the present study, we aimed to investigate the possible relationship between anger management and migraine severity (frequency and intensity of attacks) in a selected population of migrainous children/adolescents. Moreover, anxiety was also investigated in both its effect on frequency and severity of headache and its relationship with anger. We hypothesized that: 1) patients who fail to externalize their anger have higher headache frequency and intensity, 2) patients with higher anxiety scores suffer from more frequent and severe headache attacks, 3) anger expression shows a positive correlation with anxiety scores.

## Methods

### Subjects and procedures

We enrolled sixty-two (N = 62) consecutive patients suffering from migraine without aura (MoA, ICHD-II) [21] (age range 8–16 years; mean age 11.2 ± 2.1 years). They were consecutively chosen from patients referred for consultation to our Headache Centre. Twenty-nine were male (46.8%) and thirty-three were female (53.2%). MoA was diagnosed according to the criteria of the International Classification of Headache Disorders, 2nd edition (ICHD-II) [21].

At the initial visit, all patients were given a headache diary where they had to sign the main features of their headache. Data on the clinical characteristics of headache, including frequency and intensity of the attacks, were issued from the diary that was brought back at the second consultation. The attack frequency of patients suffering from episodic migraine was divided in high (HF; 10–14 attacks a month), intermediate (IF; from 5 to 9 attacks per month) and low frequency (LF; from less than once a month to 4 attacks per month). Patients who suffered from ≥ 15 attacks per month were included under the category of chronic migraine (CM). Our patients were asked to rate pain intensity on a 3-levels graduate scale: 1) mild pain (MP), allowing the subject to continue his daily activities; 2) moderate pain (MoP), leading to interruption of patient’ activities; and 3) severe pain (SP), forcing the child to go to bed.

None of the patients were receiving medications and none of them had been treated with drugs acting on the central nervous system, including drugs for migraine prophylaxis. We ensured that they did not suffer from any other neurological or internal disease.

Psychological evaluation was performed in a single session by the same examiner (S.T.) with a specific training on the psychological assessment of children and adolescents. In order to exclude a possible direct effect of pain on psychological assessment, we ensured us that no patient had a headache attack within 24 h before the psychological study.

All the patients were able to understand and to complete the tasks. None of them had ever had a previous psychological screening.

All participants and their parents gave signed, informed consent to participate to the study. The study was approved by the local ethics committee and was conducted in compliance with the Helsinki Declaration.

### Psychological tools

Psychological tools employed in our study were:

The Italian translation of the Rosenzweig Picture Frustration Study (PFS) for the anger management style [23]. It is a projective procedure to identify the patterns of response to daily stress/frustration and takes about 10–20 min to be completed. Subjects were seated in a shining and quiet room and were given a booklet including 24 black and white cartoon scenarios. These scenarios present different frustrating social situations. Each picture contains one person (P1) saying something to another person (P2) who has a blank speech box next to him/her. The patient was instructed to imagine what the P2 subject would say and to write this response in the blank speech box. Migraineurs were asked to complete the task as quickly as possible. In the present study, PFS for children (4–12 years) and for adolescents (13–18 years) were used. Both of them analyze how patients react to frustration in different situations (school, family and social) and they differ mainly in the age of the persons represented in the cartoons. Each subject’s answers to all 24 items is subsequently categorized according to nine dimensions obtained by the combination of three possible directions and of three types of aggression. Aggression may be directed: 1) toward the environment (E-extraggression), 2) toward oneself (I-intraggression), or 3) evaded (M-imaggression). There are 3 types of aggression, divided on the base of where subject’s attention is addressed: 1) focus on objects (OD-obstacle-dominance), 2) focus on people (ED-ego-defence), and 3) focus on the problem solution (NP-need-persistence). The number of times each of the 24 items is classified within the nine dimensions of anger management is the score used in the present study (Table 1). The PSF showed an inter-rater reliability ranging from 79% to 85% and demonstrates satisfactory test–retest reliability [24].

The Italian SAFA battery of tests (Psychiatric scales for self-administration for youths and adolescents) [25]. It allows examiners to explore a wide series of symptoms and psychological conditions. The entire battery includes a total of six scales (each with subscales) that can also be used separately. It evaluates anxiety-related areas (SAFA A), depression-related areas (SAFA D), obsessive–compulsive symptoms (SAFA O), somatic concerns (SAFA S), psychogenic eating disorders (SAFA P) and phobias (SAFA F). The administration can be either individual or collective (for example, screening in schools) and lasts between 30 and 60 minutes. The SAFA battery is organized to fit the mode of understanding and evaluation of a large age group: each questionnaire is in fact composed of a version for children aged 8 to 10 years (identified with the letter “e”) and a version only for subjects from 11 to 18 years (identified with the letters “ms”). Only the scale for anxiety presents three distinct versions: 8–10 years (“e”), 11–13 years (“m”) and 14–18 years (“s”). There are three possible responses to each item: ‘true, false and partly true’; the sum of points achieved in each scale and subscale can be converted into *T* scores, sten points and percentiles. On the basis of the obtained scores, it is possible to build a general profile and/or individual profiles within the single scales. The scales showed good internal consistency (Cronbach alpha > 0.80) and test-retest stability. According to the aim of our study, we administered the scale for assessment of anxiety (SAFA-A). It includes several subscales (“Generalized anxiety”, “Social anxiety”, “Separation anxiety”, “School anxiety”) and produces a “Total anxiety” score.

**Table 1:** PFS dimensions

**Direction of aggression**	**Type of aggression**		
	**Obstacle dominance (OD)**	**Ego-defence (ED)**	**Needs- persistence (NP)**
	**(The obstacle is prominent)**	**The people are prominent**	**The solution is prominent**
Extra-aggression (EA)	E’ - The presence of the obstacle frustrating is emphasized insistently.	E – Blame and hostility are directed toward a person or object in the environment.	E - A solution to the frustrating situation is requested insistently to another person.
Towards others	Example: “Stupid car!”	Example: “Great! I am going to be late because of you!”	Example: “Well! Can you call a cab then?”
Intra-aggression (IA)	I’ - The frustrating obstacle is presented as not frustrating or even beneficial; on the other hand, the subject emphasizes his/her embarrassment to be involved in causing frustration to someone else.	I – Blame and hostility are directed towards the person himself/herself.	The subject offers a repair to fix the problem because of a sense of guilt.
Towards themselves	Example: “No. It’s fine. I didn’t want to get that train anyway”	Example: “Oh no, it was my fault!”	Example: “It’s OK. I’ll pay for another thicket”.
Im-magrassion (MA)	M’ - Obstacle in the frustrating situation is minimized, almost to the point of denying its existence	M - Blame for the situation is avoided because the situation is seen as unavoidable; the person who causes frustration is absolved.	M - The subject expresses the hope that the time or circumstances lead to the solution of the problem. Patience and optimism are the main characteristics.
Neutralized/no aggression	Example: “No problem, at all”	Example: “Don’t worry, it’s not your fault that the car was broken”	Example: “Never mind. There’ll be another one soon”

### Statistical analysis

Statistical evaluation was performed using Statistical Package for the Social Sciences (SPSS 18.0) software.

According to the aim of our study, we grouped the patients based on attack frequency (LF, IF, HF and CH groups) and pain severity (MP, MoP, and SP groups). Initially, we analyzed the frequencies of each category of variables (frequency, intensity). We used descriptive statistics expressed as means, SD and percentages to describe the basic features of our sample.

Variables used in the present study are assumed to be continuous (i.e., the total- and sub- scores of SAFA or PSF dimension’ scores) or categorical (i.e., frequency of migraine attacks and intensity of pain). So non parametric correlations coefficients (Spearman coefficients) were calculated when continue-categorical combination of variables were involved. In order to use a correction for the number of tests, the Benjamini and Hochberg method [26] was applied.

To assess whether there was a relationship between the anxiety and the anger management style in our subjects, we performed a series of correlation analyses between all PFS indexes and SAFA-A subscales. In this case, since only continuous variables are involved, Pearson correlation coefficients with Bonferroni’s corrections for multiple comparisons were calculated.

A series of one-way ANOVAs were carried out to further explore differences of the anger management style and anxiety as function of the different levels of headache frequency and intensity. We compared every PFS dimensions and SAFA-A indexes between the different groups of patients. Bonferroni’s test was used for the post hoc analysis.

The level of statistical significance was set at P < 0.05.

## Results

### Headache characteristics

The frequency distribution indicated that most patients could be included in the LF group (37.1%). Fourteen patients (22.6%) suffered from high frequency attacks, while thirteen (21%) complained a chronic migraine. Patients with an intermediate attack frequency were the fewest (19.3%).

Most patients described pain as severe (41.9%); pain intensity was moderate in 30.7% and mild in 27.4% of migraineurs. Clinical characteristics of our headache patients are summarized in Table 2.

**Table 2:** Clinical characteristics of migraine patients

	**N = 62**
Pain intensity	
Mild	17 (27.4%)
Moderate	19 (30.7%)
Severe	26 (41.9%)
Frequency	
Low	23 (37.1%)
Intermediate	12 (19.3%)
High	14 (22.6%)
Chronic	13 (21.0%)
Associated symptoms	
Nausea	32 (51.6%)
Vomiting	21 (33.9%)
Phonophobia	45 (72.6%)
Photophobia	40 (64.5%)
Mean attack duration	
≤ 2 h	35 (56.4%)
> 2 h	27 (43.6%)
Headache onset	
1 year	25 (40.3%)
≤ 3 years	24 (38.7%)
> 3 years	13 (21.0%)

### Frequency of attacks, intensity of pain and anger management style

Of the nine anger management dimensions, only the IA/OD index was positively correlated with the frequency of attack (Spearman correlation r = 0.328, p = 0.041) and with the pain intensity (Spearman correlation r = 0.413, p = 0.010). Briefly, the higher the frequency of attacks and the intensity of pain the higher the tendency to direct the anger toward oneself in the attempt to dominate the problem. To further explore these relationships a series of ANOVA were conducted. ANOVA showed no difference in the main dimensions of anger direction (E, I and M) and anger type (OD, ED, NP) among the attack frequency-based groups (LF, IF, HF, and CM) (Table 3). When all dimensions were compared, our data showed a significant effect of group in PFS IA/OD dimension (failure to express anger outwardly and tendency to take the blame to be involved in causing frustration to someone else) (F(3, 58) = 3.68; p = 0.017). Bonferroni’s post hoc test revealed a difference between the LF and HF groups (p = 0.014), with the higher score for the HF group. No statistical difference was found between CM and LF patients (p = 0.288), and between IF and HF migraineurs (p = 0.568) (Table 3).

**Table 3:** PFS and SAFA-A scores (mean ± standard deviation) and ANOVA among frequency based groups

**PFS dimensions**	**Low frequency**	**Intermediate frequency**	**High frequency**	**Chronic migraine**	***F *value**	**P**
E	11.3 ± 4.7	13.4 ± 4.1	11.7 ± 5.1	10.9 ± 3.7	0.674	0.572
I	5.8 ± 2.6	4.5 ± 2.0	5.9 ± 2.6	6.7 ± 2.6	1.524	0.218
M	6.8 ± 3.3	6.0 ± 3.2	6.2 ± 3.9	6.4 ± 2.5	0.158	0.924
OD	4.8 ± 2.2	4.9 ± 1.9	5.6 ± 2.5	4.8 ± 1.9	0.510	0.677
ED	13.1 ± 2.9	12.5 ± 3.7	12.9 ± 3.5	12.9 ± 2.9	0.072	0.975
NP	5.9 ± 3.1	6.6 ± 3.7	5.3 ± 1.9	6.3 ± 3.0	0.432	0.731
EA/OD	3.1 ± 1.9	2.4 ± 1.7	2.8 ± 1.9	2.1 ± 1.7	0.831	0.482
EA/ED	6.2 ± 3.7	7.8 ± 3.0	6.8 ± 4.3	6.6 ± 3.4	0.533	0.654
EA/NP	2.1 ± 1.6	3.0 ± 2.6	2.7 ± 3.3	2.3 ± 1.6	0.505	0.680
IA/OD	0.3 ± 0.4	0.5 ± 0.6	1.0 ± 1.1	0.8 ± 0.8	3.679	0.017*
IA/ED	3.9 ± 1.8	2.5 ± 0.8	3.3 ± 2.4	3.7 ± 1.7	1.623	0.194
IA/NP	1.6 ± 1.6	1.5 ± 1.5	1.6 ± 1.6	2.2 ± 1.7	0.514	0.675
MA/OD	1.7 ± 1.2	1.9 ± 1.8	2.5 ± 2.9	2.1 ± 1.5	0.562	0.642
MA/ED	2.9 ± 2.1	2.1 ± 1.5	3.5 ± 3.8	2.6 ± 1.6	0.712	0.548
MA/NP	2.2 ± 1.6	2.0 ± 1.4	2.2 ± 1.8	2.8 ± 0.9	0.220	0.882
**SAFA-A**	**Low frequency**	**Intermediate frequency**	**High frequency**	**Chronic migraine**	***F *value**	**P**
SAFA-A Ge (Generalized anxiety)	10.5 ± 4.9	10.9 ± 5.5	9.4 ± 4.9	8.8 ± 4.3	0.527	0.666
SAFA-A So (Social anxiety)	8.1 ± 3.4	8.5 ± 3.1	6.0 ± 3.4	8.0 ± 4.3	1.398	0.253
SAFA-A Se (Separation anxiety)	9.4 ± 5.0	6.7 ± 5.4	7.6 ± 4.1	3.3 ± 3.5	5.229	0.003*
SAFA-A Sc (School anxiety)	10.3 ± 4.7	9.3 ± 5.9	9.0 ± 4.3	8.3 ± 4.7	0.553	0.648
SAFA-A Tot (Total anxiety)	38.2 ± 14.0	35.4 ± 17.4	32.0 ± 15.4	28.4 ± 12.2	1.412	0.248

For the meaning of the PFS dimensions, see Table 1.

*P ≤ 0.05.

When pain severity was examined, no significant effect of pain intensity-based groups (MP, MoP, and SP) for the main dimensions of anger direction (E, I and M) and anger type (OD, ED, NP) was found. However, a significant difference was found for the IA/OD dimension. (F(2, 59) = 6.78; p = 0.002). SP patients showed a higher IA/OD value than MP (p = 0.008) and MoP (p = 0.011) patients (Table 4).

**Table 4:** PFS and SAFA-A scores (mean ± standard deviation) and ANOVA among pain intensity based groups

**PFS dimensions**	**Low frequency**	**Intermediate frequency**	**High frequency**	***F *value**	**P**
E	12.4 ± 4.3	11.1 ± 4.0	11.7 ± 4.9	0.375	0.689
I	5.2 ± 2.5	5.7 ± 2.5	6.2 ± 2.6	0.788	0.460
M	6.3 ± 3.1	7.0 ± 3.4	6.0 ± 3.2	0.535	0.589
OD	5.2 ± 1.8	4.4 ± 2.2	5.3 ± 2.3	0.920	0.404
ED	13.4 ± 2.1	13.3 ± 2.9	12.2 ± 3.8	0.979	0.382
NP	5.2 ± 2.6	5.9 ± 2.3	6.5 ± 3.4	1.088	0.344
EA/OD	3.1 ± 1.7	2.4 ± 1.8	2.6 ± 1.9	0.693	0.504
EA/ED	6.8 ± 3.5	6.6 ± 2.9	6.7 ± 4.3	0.007	0.993
EA/NP	2.5 ± 2.3	2.6 ± 2.9	2.4 ± 1.6	0.490	0.952
IA/OD	0.3 ± 0.5	0.3 ± 0.5	1.0 ± 0.9	6.780	0.002*
IA/ED	3.8 ± 2.1	3.9 ± 1.7	3.1 ± 1.8	1.279	0.286
IA/NP	1.1 ± 1.2	1.5 ± 1.6	2.2 ± 1.7	2.624	0.081
MA/OD	1.9 ± 1.4	1.9 ± 1.7	2.0 ± 2.3	0.420	0.959
MA/ED	2.9 ± 2.4	3.3 ± 3.0	2.5 ± 1.9	0.686	0.508
MA/NP	1.5 ± 0.8	2.4 ± 1.3	2.2 ± 1.9	1.418	0.250
**SAFA-A**	**Low frequency**	**Intermediate frequency**	**High frequency**	***F *value**	**P**
SAFA-A Ge (Generalized anxiety)	8.8 ± 4.7	11.1 ± 4.9	9.8 ± 4.9	1.086	0.344
SAFA-A So (Social anxiety)	7.9 ± 3.4	6.8 ± 3.2	8.2 ± 4.1	0.808	0.451
SAFA-A Se (Separation anxiety)	7.8 ± 5.4	7.2 ± 4.6	6.9 ± 4.9	0.167	0.847
SAFA-A Sc (School anxiety)	9.0 ± 4.7	10.6 ± 3.8	8.8 ± 5.5	0.803	0.453
SAFA-A Tot (Total anxiety)	33.6 ± 14.9	35.7 ± 13.9	33.7 ± 15.8	0.121	0.886

*P ≤ 0.05.

For the meaning of the PFS dimensions, see Table 1.

In conclusion, we found a relationship between IA/OD dimensions and both attack intensity and frequency. Children with a high attack frequency and those who complained the highest intensity showed the tendency to inhibit anger expression. In particular, they experienced the frustrating obstacle as not frustrating or considered themselves responsible of the problem.

### Frequency of attacks, intensity of pain and anxiety

Attack frequency, but not the intensity, showed a significant effect on anxiety symptoms. In particular, a negative and significant correlation emerged between Separation Anxiety (SAFA-ASE) and the frequency of attacks (Spearman correlation r = −0.409, p = 0.006). When all subscales were compared by ANOVA, it was the “Separation anxiety” subscale score to show a significant intergroup difference (F(3, 58) = 5.22; p = 0.003) (Table 3). LF group had higher “Separation anxiety” scores than CM patients (p = 0.001). Pain intensity did not show any significant effect on SAFA-A subscales (Table 4).

Our data showed a relationship between the separation anxiety symptoms and the frequency of migraine attacks. In particular, children with a lower attack frequency had higher anxiety scores.

### Relationship between anger management style and anxiety

Furthermore in our patient’s anger management/expression style showed a relationship with anxiety symptoms. In particular, the tendency to express and emphasize the presence of the frustrating obstacle (EA/OD) could increase anxiety level. Analyzing all PFS dimensions, we found a significant and positive correlation between EA/OD scores (the presence of the frustrating obstacle is insistently emphasized) and the “Separation anxiety” (r = 0.402; p = 0.008) subscales and with “Total anxiety” (r = 0.345; p = 0.033) scales.

## Discussion

This is the first study to analyze the possible correlation between anger management style (both direction and type), anxiety and headache features in a selected population of children/adolescents with migraine. The main results of the present study are:

(1) there is a relationship in terms of correlations and of mean differences between both attack frequency and intensity and failure to express the anger feelings outwardly,

(2) unlike what we expected, children with a low frequency of attacks have higher “Separation anxiety”,

(3) there are correlations between the anger expression/management style and anxiety disorders.

### Headache severity and anger management

Among our patients, children with higher attack frequency and intensity had the tendency to inhibit anger expression, that is to appear as if they considered the frustrating obstacle as not frustrating or to blame themselves (IA/OD index) to cause frustration to someone else.

That in our study only the HF patients, but not the CM migraineurs, were significantly different in the IA/OD index from the LF group is difficult to explain. We can hypothesize that, as suggested by several studies [27,28], the pathophysiological mechanisms of pain in chronic migraineurs are different from those in patients with episodic migraine. This can make it difficult to compare CM subjects with patients with episodic migraine. A further hypothesis is that the relationship between headache severity and anger management is not linear, but it increases in the passage between LF and HF and then it reaches a plateau. As a consequence, no more differences can be found between HF and CM patients.

Very few studies analyzed the role of anger in the headache frequency and intensity [13,15-17] and only one study dealt with children [20]. In adult patients, no significant correlation was found between the anger management style and both headache frequency and intensity [13,16,17]. In children, Kristjándóttir found that young headache sufferers with higher attack frequency were those who showed also higher anger levels [20]. The disagreement between these previous results and our data, showing worse headache frequency in patients with lower anger scores, can be due to 3 main elements: 1) Kristjándóttir’s study was a part of a large investigation of self-report pain in school children and did not focus on patients suffering from migraine; 2) headache diagnosis was performed by a self report questionnaire and was not based on the ICHD-II criteria [21]; 3) the author did not use a standardized test to assess the main components of anger.

### Headache and anxiety

In the present study, Separation anxiety subscale showed the highest scores in the migraineurs with a low attack frequency. Separation anxiety disorder (SAD) is an anxiety disorder of middle childhood characterized by an excessive worry about separation from another person who represents safety for the affected child, typically a parent [29,30]. Somatic symptoms such as headache, nausea and more often abdominal pain are common features of SAD [29,31]. They often occur in the context of separation situation, reflecting either an avoidance strategy or genuine physical distress. Children and adolescents who have insecure models of attachment may display exaggerated nonverbal affective signals such as anger, fear, somatic complaints or desire for comfort, thereby eliciting more predictable responses from parents [32,33]. Previous research has shown that these children report high pain intensity and disability [34,35]. These previous results seem in disagreement with our present findings showing that the lower the attack frequency was, the worse the SAD the child could experience. This can be explained by the fact that previous studies did not deal with migraine [31,34-36]. Insecure attachment represents a predictor for higher disability in adult migraineurs [37,38]. To our knowledge, there are not studies investigating the possible relationship between attachment and headache in pediatric age. Although we did not investigate the attachment style with specific psychological tools, our results suggest that in children attachment oriented to the proximity may reduce headache severity.

### Relationship between anxiety and anger management

While the relationship between anger expression and depression has been repeatedly investigated in headache [12,14-17] and in chronic pain [39], possible correlations between anger and anxiety have been rarely explored and all studies referred to adulthood [12,16]. The current study, performed on a pediatric headache population, confirmed the relationship between anger and anxiety described in adult populations [16]. In our patients, anger directed outwards (EA/OD index) was associated with separation and school anxiety. Anger is a negative affect and anxiety may depend on overt expression of anger, which could make the individual less lovable. This can be explained, considering that perceived consequences of anger may include fear of being “unacceptable” by parents who usually are the most important attachment figures in pediatric age [40].

### Limitations of the study

The present study has some limitations that have to be underlined. 1) The explored relationships have a correlational character. Further prospective longitudinal studies need to be conducted in order to ascertain if there exist a causal relationship between the variables involved. 2) Our results were issued from children who were clinically referred to our third-level centre for the treatment of primary headache. This may represent a selection bias with regard to the whole population of patients suffering from migraine without aura. 3) The study was a pilot study and it did not include a group of healthy controls. Future studies involving a larger sample size and the presence of a control group are required to draw definitive conclusions. 4) Limitations of the psychological tools have also to be addressed. The SAFA-A test, used to investigate anxiety, has a fundamental self-report nature. Concerning the PFS, the validity of the aggressiveness measures might be hampered by social desirability and self-presentational concerns about a socially unacceptable behaviour as aggression.

## Conclusions

This is the first study to analyze the relationship between anger, anxiety and headache in a paediatric population. We found that children suffering from severe migraine tend to inhibit their angry feelings. Moreover, anger is associated with anxiety symptoms (separation anxiety), which, on their turn, are typical of migraineurs with a low attack frequency. In conclusion, our results suggest that children with worse migraine symptoms (intensity and frequency of the attacks) are those who less often express their anger. On the contrary, children with low migraine attack frequency express their anger and suffer from separation anxiety.

Future studies will have to test whether these psychological features can be important in migraine therapeutical management.

## Competing interests

The authors declare that they have no competing interests.

## Authors’ contributions

ST and MV conceived and supervised the project, drafted the manuscript and were the main authors. ST, CDR and SG were responsible for data collection. CD and FP were involved in data analysis and interpretation and assisted in preparation of the manuscript. MC, AC, FG, VG and FG critically reviewed and revised this manuscript for important intellectual inputs. All authors read and approved the manuscript.

## References

[B1] Nicholson RA Houle TT Rhudy JL Norton PJ Psychological risk factors in headache Headache 2007 14 3 413 426 review 17371358 10.1111/j.1526-4610.2006.00716.x PMC2408884

[B2] Guidetti V Galli F Psychiatric comorbidity in chronic daily headache: pathophysiology, etiology, and diagnosis Curr Pain Headache Rep 2002 14 6 492 497 review 10.1007/s11916-002-0069-7 12413409

[B3] Antonaci F Nappi G Galli F Manzoni GC Calabresi P Costa A Migraine and psychiatric comorbidity: a review of clinical findings J Headache Pain 2011 14 2 115 125 review 10.1007/s10194-010-0282-4 21210177 PMC3072482

[B4] Powers SW Gilman DK Hershey AD Headache and psychological functioning in children and adolescents Headache 2006 14 9 1404 1415 review 10.1111/j.1526-4610.2006.00583.x 17040337

[B5] Fernandez E Turk DC The scope and significance of anger in the experience of chronic pain Pain 1995 14 2 165 175 10.1016/0304-3959(95)00192-U 7659426

[B6] Kerns RD Rosenberg R Jacob MC Anger expression and chronic pain J Behav Med 1994 14 1 57 67 10.1007/BF01856882 8201612

[B7] Burns JW Johnson BJ Devine J Mahoney N Pawl R Anger management style and the prediction of treatment outcome among male and female chronic pain patients Behav Res Ther 1998 14 11 1051 1062 10.1016/S0005-7967(98)00080-1 9737057

[B8] Okifuji A Turk DC Curran SL Anger in chronic pain: investigations of anger targets and intensity J Psychosom Res 1999 14 1 1 12 10.1016/S0022-3999(99)00006-9 10511417

[B9] Sayar K Gulec H Topbas M Alexithymia and anger in patients with fibromyalgia Clin Rheumatol 2004 14 5 441 448 10.1007/s10067-004-0918-3 15278756

[B10] Thomas E Moss-Morris R Faquhar C Coping with emotions and abuse history in women with chronic pelvic pain J Psychosom Res 2006 14 1 109 112 10.1016/j.jpsychores.2005.04.011 16380318

[B11] Spielberger CD Johnson EH Russell SF Crane RJ Jacobs GA Worden TI Chesney MA, Rosenman RH The experience and expression of anger: construction and validation of an anger expression scale Anger and hostility in cardiovascular and behavioral disorders 1985 New York: Hemisphere

[B12] Nicholson RA Gramling SE Ong JC Buenaver L Differences in anger expression between individuals with and without headache after controlling for depression and anxiety Headache 2003 14 6 651 663 10.1046/j.1526-4610.2003.03108.x 12786926

[B13] Perozzo P Savi L Castelli L Valfrè W Lo Giudice R Gentile S Raniero I Pinessi L Anger and emotional distress in patients with migraine and tension-type headache J Headache Pain 2005 14 5 392 399 10.1007/s10194-005-0240-8 16362712 PMC3452065

[B14] Abbate-Daga G Fassino S Lo Giudice R Rainero I Gramaglia C Marech L Amianto F Gentile S Pinessi L Anger, depression and personality dimensions in patients with migraine without aura Psychother Psychosom 2007 14 2 122 128 10.1159/000097971 17230053

[B15] Materazzo F Cathcart S Pritchard D Anger, depression, and coping interactions in headache activity and adjustment: a controlled study J Psychosom Res 2000 14 1 69 75 10.1016/S0022-3999(00)00144-6 11053606

[B16] Venable VL Carlson CR Wilson J The role of anger and depression in recurrent headache Headache 2001 14 1 21 30 10.1046/j.1526-4610.2001.111006021.x 11168600

[B17] Tschannen TA Duckro PN Margolis RB Tomazic TJ The relationship of anger, depression, and perceived disability among headache patients Headache 1992 14 10 501 503 10.1111/j.1526-4610.1992.hed3210501.x 1468908

[B18] Odegaard G Lindbladh E Hovelius B Children who suffer from headaches: a narrative of insecurity in school and family Br J Gen Pract 2003 14 488 210 213 14694697 PMC1314546

[B19] Lanzi G Zambrino CA Ferrari-Ginevra O Termine C D’Arrigo S Vercelli P De Silvestri A Guglielmino CR Personality traits in childhood and adolescent headache Cephalalgia 2001 14 1 53 60 10.1046/j.1468-2982.2001.00144.x 11298664

[B20] Kristjándóttir G The relationship between pains and various discomforts in school children Childhood 1997 14 4 491 504 10.1177/0907568297004004008

[B21] Headache Classification Committee of International Headache Society Classification and diagnostic criteria for headache disorders, cranial neuralgias and facial pain, 2nd edition Cephalalgia 2004 14 Suppl 1 1 160 3048700

[B22] Bihldorff JP King SH Parnes LR Psychological factors in headache Headache 1971 14 117 127 10.1111/j.1526-4610.1971.hed1103117.x 5115598

[B23] Rosenzweig S PFS-Picture frustration study. Lo studio della reazione alle frustrazioni Lo studio della reazione alla frustrazione 1992 Firenze: Lo studio della reazione alle frustrazioni Organizzazioni Speciali

[B24] Rosenzweig S Ludwig DJ Adelman S Retest reliability of the rosenzweig picture-frustration study and similar semiprojective techniques J Pers Assess 1975 14 1 3 12 10.1207/s15327752jpa3901_1 16367414

[B25] Cianchetti C Sannio Fascello G Scale psichiatriche di autosomministrazione per fanciulli e adolescenti (SAFA) 2001 Firenze: Organizzazioni Speciali

[B26] Benjamini Y Hochberg Y Controlling the false discovery rate: a practical and powerful approach to multiple testing J Roy Stat Soc Ser B 1995 14 289 300

[B27] Ferraro D Vollono C Miliucci R Virdis D De Armas L Pazzaglia C Le Pera D Tarantino S Balestri M Di Trapani G Valeriani M Habituation to pain in “medication overuse headache”: a CO2 laser-evoked potential study Headache 2012 14 5 792 807 10.1111/j.1526-4610.2012.02151.x 22512411

[B28] Coppola G Schoenen J Cortical excitability in chronic migraine Curr Pain Headache Rep 2012 14 1 93 100 10.1007/s11916-011-0231-1 22076672

[B29] American Psychiatric Association Diagnostic and statistical manual of mental disorders (4th edn-text revision) 2000 Washington, DC: Task Force

[B30] Ehrenreich JT Santucci LC Weiner CL Separation anxiety disorder in youth: phenomenology, assessment, and treatment Psicol Conductual 2008 14 3 389 412 19966943 10.1901/jaba.2008.16-389 PMC2788956

[B31] Walker LS Beck J Anderson J Functional abdominal separation anxiety: helping the child return to school Pediatr Ann 2009 14 5 267 271 19476299 PMC3205969

[B32] Kozlowska K Attachment relatioships shape pain-signaling behavior J Pain 2009 14 10 1020 1028 review 10.1016/j.jpain.2009.03.014 19556169

[B33] Bowlby J Attachment and Loss 1973 14 New York: Basic Book

[B34] Walsh TM McGrath PJ Symons DK Attachment dimentions and young children’s response to pain Pain Res Manag 2008 14 1 33 40 18301814 10.1155/2008/235329 PMC2670808

[B35] Tremblay I Sullivan MJ Attachment and pain outcomes in adolescents: the mediating role of pain catastrophizing and anxiety J Pain 2010 14 2 160 171 10.1016/j.jpain.2009.06.015 19853522

[B36] Puskar KR Sereika SM Haller LL Anxiety, somatic complaints, and depressive symptoms in rural adolescents J Child Adolesc Psychiatr Nurs 2003 14 3 102 111 10.1111/j.1744-6171.2003.00102.x 14603986

[B37] Rossi P Di Lorenzo G Malpezzi MG Di Lorenzo C Cesarino F Faroni J Siracusano A Troisi A Depressive symptoms and insecure attachment as predictors of disability in a clinical population of patients with episodic and chronic migraine Headache 2005 14 5 561 570 10.1111/j.1526-4610.2005.05110.x 15953275

[B38] Savi L Buccheri R Tambornini A De Martino P Albasi C Pinessi L Attachment styles and headache J Headache Pain 2005 14 4 254 257 10.1007/s10194-005-0200-3 16362679 PMC3452001

[B39] Burns JW Quartana PJ Bruehl S Anger inhibition and pain: conceptualizations, evidence and new directions J Behav Med 2008 14 3 259 279 review 10.1007/s10865-008-9154-7 18498056

[B40] Waldinger RJ Schulz MS Barsky AJ Ahern DK Mapping the road from childhood trauma to adult somatization: the role of attachment Psychosom Med 2006 14 1 129 135 10.1097/01.psy.0000195834.37094.a4 16449423

